# Chinese medicine combined with calcipotriol betamethasone and calcipotriol ointment for Psoriasis vulgaris (CMCBCOP): study protocol for a randomized controlled trial

**DOI:** 10.1186/1745-6215-15-294

**Published:** 2014-07-22

**Authors:** Ze-Huai Wen, Mei-Ling Xuan, Yu-Hong Yan, Xiao-Yan Li, Dan-Ni Yao, Geng Li, Xin-Feng Guo, Ai-Hua Ou, Chuan-Jian Lu

**Affiliations:** 1 Key Unit of Methodology in Clinical Research, Guangdong Provincial Hospital of Chinese Medicine, 111 Dade Road, Guangzhou 510120, China; 2 National Centre for Design Measurement and Evaluation in Clinical Research, Guangzhou University of Chinese Medicine, 12 Jichang Road, Guangzhou 510405, China; 3 Department of Dermatology, Guangdong Provincial Hospital of Chinese Medicine, 111 Dade Road, Guangzhou 510120, China

**Keywords:** Psoriasis vulgaris, Randomized controlled trial, Calcipotriol Betamethasone, Calcipotriol, YXBCM01 granule, Chinese medicine

## Abstract

**Background:**

Psoriasis causes worldwide concern because of its high-prevalence, as well as its harmful, and incurable characteristics. Topical therapy is a conventional treatment for psoriasis vulgaris. Chinese medicine (CM) has been commonly used in an integrative way for psoriasis patients for many years. Some CM therapies have shown therapeutic effects for psoriasis vulgaris (PV), including relieving symptoms and improving quality of life, and may reduce the relapse rate. However, explicit evidence has not yet been obtained. The purpose of the present trial is to examine the efficacy and safety of the YXBCM01 granule, a compound Chinese herbal medicine, with a combination of topical therapy for PV patients.

**Methods/Design:**

Using an add-on design, the trial is to evaluate whether the YXBCM01 granule combined topical therapy is more effective than topical therapy alone for the treatment of PV. The study design is a double-blind, parallel, randomized controlled trial comparing the YXBCM01 granule (5.5 g twice daily) to a placebo. The duration of treatment is 12 weeks. A total of 600 participants will be randomly allocated into two groups, YXBCM01 granule group and placebo group, from 11 general or dermatological hospitals in China. Topical use of calcipotriol betamethasone for the first 4 weeks and calcipotriol ointment for the remaining 8 weeks will be the same standard therapy for the two groups. Patients will be enrolled if they have a clinical diagnosis of PV, a psoriasis area severe index (PASI) of more than 10 or body surface area (BSA) of more than 10%, but PASI of less than 30 and BSA of less than 30%, are aged between 18 and 65-years-old, and provide signed informed consent. The primary outcome, relapse rate, is based on PASI assessed blindly during the treatment. Secondary outcomes include: (i) relapse time interval, (ii) time to onset, (iii) rebound rate, (iv) PASI score, (v) cumulative consumption of medicine, (vi) the dermatology quality life index (DLQI), and (vii) the medical outcomes study (MOS) item short form health survey (SF-36). Analysis will be on intention-to-treat and per-protocol subject analysis principles.

**Discussion:**

To address the effectual remission of the YXBCM01 granule for PV, this trial may provide a novel regimen for PV patients if the granule can decrease relapse rate without more adverse effects.

**Trial registration:**

Chinese Clinical Trial Registry (http://cwww.chictr.org): ChiCTR-TRC-13003233, registered 26 May 2013.

## Background

Psoriasis is an immune-abnormal, chronic, proliferative skin disease determined by polygenic inheritance and induced by a number of environmental factors. The prevalence rates of psoriasis in Europe vary between 0.73% (in Scotland) and 2.9% (in Italy), and rates reported in United States vary between 0.7% and 2.6%, which shows a worldwide geographic variation [1]. In China, the incidence of psoriasis has increased to 0.47% [2], showing an upward trend of 0.12% compared to 1984 [3]. Psoriasis is characterized by scaly, erythematous patches, papules, and plaques that are often severely pruritic. Symptoms such as poor esteem, sexual dysfunction, anxiety, depression, and suicidal ideation due to the appearance of skin, can significantly reduce the patients’ health-related quality of life. For instance, in China, 59.8% of psoriatic people experience negative influences on quality of life [2]. It has been a cause of increasing concern worldwide due to its high-prevalence and its harmful and incurable characteristics.

Topical therapies remain the primary method of managing mild to moderate psoriasis, including topical corticosteroids, vitamin D analogues, sulfur ointment, laser therapy, dithranol, tazarotene, and coal tar [4,5]. In particular situations, plaque psoriasis, erythrodermic psoriasis, generalized pustular psoriasis, and refractory cases are treated with systematic therapy such as retinoic acid, methotrexate, glucocorticoid, and biologicals. However, most of the systematic therapies have serious side effects and are an expensive option, thus limiting their long-term use [6].

Over the years, Chinese medicine (CM) for treatment of psoriasis has accumulated a wealth of clinical experience. Some CM therapies have shown long lasting therapeutic effects on controlling psoriasis vulgaris (PV) with minimal side effects. CM can also alleviate the symptoms effectively, reduce the recurrence rate, and control the condition with fewer side effects in psoriasis treatment [7-9]. A systematic review of CM for psoriasis treatment, including 32 randomized controlled trials worldwide, reporting 5,179 patients with PV, suggested that some certain CM interventions or combining CM with conventional medicine had promising results for PV [10]. The topical herbal formula and herbal medicines adding to conventional therapy for psoriasis appears to have potential benefits for symptom reduction and is also relatively safe [11,12]. However, there was previously no evidence supporting CM as an effective and safe therapy against PV relapse.

A Chinese herbal compound, YXBCM01 formula, is theorized to have an effect on reducing PV’s relapse rate based on Chinese medicine theory and clinical observations. This formula composed of *Radix Paeoniae Rubra* (Chishao), *Sarcandra Glabra* (Jiujiecha), *Rhizoma Smilacis Glabrae* (Tufuling) and more, and was developed by Professor Xuan Guowei, a well-known CM dermatologist in China. YXBCM01 is the most commonly used formula to treat PV in Guangdong Provincial Hospital of Chinese Medicine (GPHCM) for over 20 years, and has been registered with the Guangdong Food and Drug Administration (Hospital preparation approval number Z20080123). An observational study showed two months treatment of YXBCM01 for PV reduced Psoriasis Area Severe Index (PASI) and Dermatology Life Quality Index (DLQI) scores, moreover, no adverse reaction was reported during the study period [13].

According to the German guidelines on treatment of PV, topical therapy with a combination of calcipotriol betamethasone for the first 4 weeks and calcipotriol ointment for the remaining 8 weeks is recommended, since it was significantly superior from a pharmacoeconomic standpoint when compared to the use of each therapy alone [13]. Therefore, a randomized, double-blind, placebo-controlled add-on study is designed to evaluate the efficacy of YXBCM01 concurrent with topical therapy of calcipotriol and betamethasone in reducing the relapse rate of PV. Results of this study will provide evidence regarding the value of YXBCM01 as an intervention for PV patients.

## Method/Design

### Design and setting

**Figure 1:**
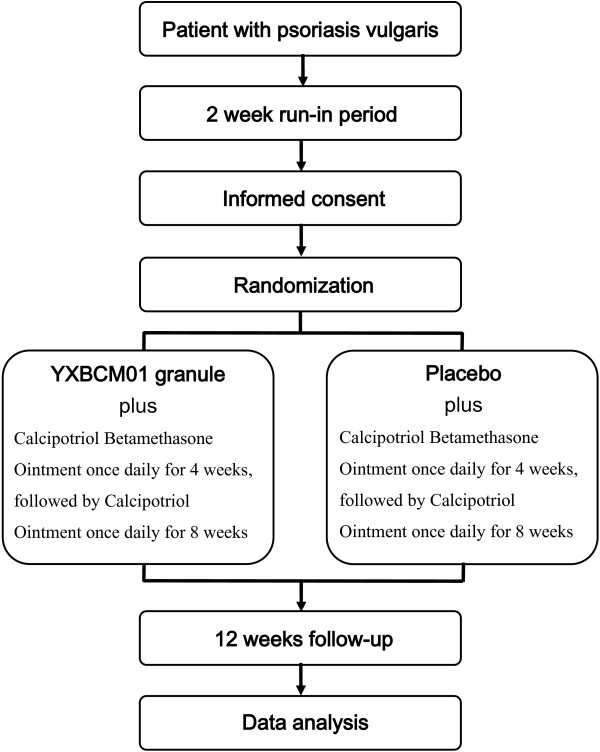
CMCBCOP trial flow diagram.

This is a multicenter, randomized controlled trial, which will be conducted in 11 general or dermatological hospitals in different provinces in China. The study aims to enroll 600 patients with PV over a three-year period. Participants are randomized using a ratio of 1:1 to receive either an oral YXBCM01 granule (5.5 g twice daily) or a placebo. After a 2 to 4 week run-in period for the screening of subjects and providing written informed consent, patients will be randomly allocated into two groups to undergo a treatment period of 12 weeks. A follow-up period of 12 weeks will continue until the study close (see Figure 1 and Additional file 1).

This trial was approved by six sites’ ethics committees and required archival filing management in five other sites’ ethics committees (Additional file 2).

### Participants

Patients with a clinical diagnosis of psoriasis plaque (as diagnosed by a dermatologist during the run-in period) can be enrolled into this study. A diagnosis of PV is compliant with the guidelines of care for the management of psoriasis and psoriatic arthritis from the American Academy of Dermatology and the guideline for the treatment of psoriasis from the Psoriasis Study Group of Chinese Medical Association [14,15].

Research assistants will introduce and discuss the trial to potential subjects using Mandarin or the local language. All potential subjects will be given a consent form and separate information sheets including information on the main aspects of the trial. Patients will then be able to have an informed discussion with their family and participating consultant. Research assistants will obtain the signed consent form from patients willing to participate in the trial.

#### Recruitment procedure

Most participants will be recruited through posters in local newspapers and hospitals; a few patients may spontaneously contact trial centers.

#### Inclusion criteria

Patients will be enrolled if they have a clinical diagnosis of PV, a psoriasis area severe index (PASI) of more than 10 or body surface area (BSA) of more than 10%, but PASI of less than 30 and BSA of less than 30%, are aged between 18 and 65-years-old, and provide signed informed consent.

#### Exclusion criteria

1. Those with guttate psoriasis, inverse psoriasis or psoriasis exclusively involving the face

2. Those who are pregnant, lactating, or who plan to become pregnant within a year

3. A Self-rating Anxiety Scale (SAS) score of > 50 or a Self-rating Depression Scale (SDS) scale of > 53, or those with other psychiatric disorders

4. Those with any uncontrolled cardiovascular, respiratory, digestive, urinary, or hematological disease

5. Those with a known cancer, infection, electrolyte imbalance, acid-base disturbance or calcium metabolic disorder

6. An abnormal serum calcium level (Ca^2+^ > 2.9 mmol/L or < 2 mmol/L)

7. Anyone allergic to any medicine or ingredients used in this study

8. Anyone currently enrolled in other clinical trials or who have participated in another trial within a month

9. Anyone who has received topical treatments (such as corticosteroids or retinoic acid) within 2 weeks, systemic therapy or phototherapy (ultraviolet radiation B,UVB and psoralen combined with ultraviolet A, PUVA) within 4 weeks, or biological therapy within 12 weeks

10. Acute progression of psoriasis and erythroderma tendency

11. Patients need systemic treatment prescribed by doctors

### Interventions

Participants in experimental group will receive the YXBCM01 granule 5.5 g twice daily for 12 weeks. Placebo granule are given to patients in the placebo group 5.5 g twice daily for 12 weeks, the main ingredients of which are maltodextrin, lactose, and a natural edible pigment, will be identical to the YXBCM01 granule in appearance, weight, and taste. The sequential topical therapy is administrated simultaneously in all eligible patients by using a calcipotriol betamethasone ointment once daily (treatment area up to 30% BSA, one fingertip unit is recommended) in the first 4 weeks (maximum of 100 g weekly), followed by calcipotriol ointment once daily in the remaining 8 weeks (maximum of 100 g weekly), which is recommended on the S3-Guidelines for the treatment of psoriasis vulgaris [5]. The YXBCM01 granule and the matching placebo used in the trial are manufactured by the Tianjiang Pharmaceutical Co., Ltd. (Jiangyin, Jiangsu Province, China) that meets the requirements of the Good Manufacturing Practice (GMP). The calcipotriol betamethasone ointment (approval number H20080487) and calcipotriol ointment (approval number H20050125) are manufactured by LEO Laboratories Ltd (Ballerup, Denmark).

#### Rescue and concomitant treatment

Urea ointment is used as the basic concomitant treatment in the run-in and follow-up period, according to doctors’ opinion. In cases of patients with serious itch, cetirizine hydrochloride (1 pill per day) is to be a rescue drug following the doctor’s advice.

### Randomization and blinding

A total of 600 participants will be enrolled from eligible patients in 11 research sites. A computer-generated random list for center-stratified method and permuted blocks size created by SAS 9.2 software (SAS Institute Inc., Cary, USA) and performed by the Key Unit of Methodology in Clinical Research (KUMCR) of Guangdong Provincial Hospital of Chinese Medicine will be used for randomization. The participants will be randomly allocated to two different treating groups in a 1:1 ratio through the Interactive Web Response System for Chinese Medicine Trials (IWRS-CMT), which was a verified online randomization facility established by the KUMCR.

The randomization schedule will be concealed until interventions are all assigned and enrollment, follow-up, data collection, data cleaning, and analysis are complete. The participants, paramedics, investigators, outcomes assessors, statistician, and other staff do not know the treatment allocation, which will not be revealed until the end of study.

The randomization list and blinding codes will be kept strictly confidential. Only the KUMCR staff will have access to the randomization list. Blinding was ensured using a matched placebo granule identical in color, size, shape, and taste. The quality of the matched trial supplies, such as contents, solubility, and bacteria contaminations, should be controlled rigorously according to the GMP standards, and be tested and verified by researchers.

### Outcome measures

The primary outcome measure in the trial is relapse incidence rate in the treatment period and follow-up period. The definition of relapse is a loss of 50% of PASI improvement from baseline in patients who have achieved treatment success (at least 50% improvement in PASI score from baseline) [16,17]. PASI will be assessed every week during the first 4 weeks and every 2 weeks throughout the rest of the treatment and follow-up period. Meanwhile, patients will be required to report the emergency of skin lesions at any time in the study period, and researchers will assess the PASI score on the same or closest day.

Secondary outcome measures include time to relapse, time to onset, rebound rate, PASI score, cumulative consumption of topical medicine, visual analogue scale (VAS), BSA, DLQI, and SF-36 (the MOS item short form health survey).

Time to relapse is defined as the time to loss of at least 50% of the PASI improvement for the first time. Time to onset is time taken for the PASI score to decrease more than 50% for the first time, and treatment will be considered as ineffective when the PASI score cannot get 50% improvement throughout the treatment period. Rebound refers to a PASI score increasing of more than 125% above baseline, or occurring of new generalized pustular and erythrodermic after its improvement (PASI-50) during the study period. The VAS and BSA will be assessed every week during the first 4 weeks and every 2 weeks throughout the rest of the treatment period. The DLQI and SF-36 will be self-assessed by patients every 4 weeks throughout this trial.

### Safety assessments

Participants are to be questioned and report all adverse events (AEs) at each visit point, and all AEs reports will be recorded and assessed by the investigators. A blood test, urinalysis, hemagglutination test, and electrocardiograph examination will be checked before and after the treatment. Furthermore, calcium, kidney, and liver function tests will be reexamined at week 4 and week 12, respectively. All abnormal changes from the baseline of lab test will be evaluated.

### Sample size calculation

Based on White *et al*.’s study [18], the relapse rate of sequential topical therapy 12 weeks is 37.3%, and placebo is 46.6%. For example, suppose the relapse rate of YXBCM01 granule combined with sequential topical therapy for PV in the twelfth week is 20%, sequential topical therapy alone is 37%. According to the supposition and as calculated by PASS 11.0 software (NCSS, LLC, Kaysville, Utah, USA), sample size of 239 in each group can achieve 90% power and to rule out a two-sided type I error of 5% to detect a superiority margin difference of 10% in this two-arm trial with equal allocation in each group. Considering 15 to 20% loss to follow-up, the total sample size should be adjusted to 300 in each group.

### Data management and quality control

The data collected in this trial comprises of information recorded in case report forms and information on the DLQI and SF-36 scale. When every visit was completed at each center, data will be entered using the double entry method.

To ensure that outcome assessments are of a high standard in accordance with the trial protocol, physicians, assessors, and research assistants will attend six-hours training workshop before the conduction of trial. They will also be provided with a written protocol and standard operation procedures documents. All the data in different sites will be checked regularly by researchers from GPHCM and be overseen by monitors. The monitoring tasks of the trial will be entrusted to Guangdong International Clinical Research Center of Chinese Medicine (Guangzhou, China). The auditing and inspection of the trial will be performed by the Department of Science Research of GPHCM and the Office of National Key Technology R&D Program for the Twelfth Five-year Plan of Ministry of Science and Technology, China. The Data Monitoring Committee from GPHCM will assess the safety data and the critical efficacy outcomes.

Participants may withdraw from the study at any time for any reason. If any patients wish to withdraw, clinicians should ask if they would be willing to complete the assessments as according to the study schedule and write down their last time of taking the medicine. Incidences of patient loss to follow-up and withdrawal will be recorded and reported.

### Statistical analysis

Data analysis will follow with the statistical analysis plan for this trial. Data will be processed by statistical analyses with PASW Statistics 18.0 (IBM SPSS Inc., Armonk, New York, USA) and SAS 9.2 software (SAS Institute Inc., Cary, USA). Two-tailed *P* values <0.05 are considered to be statistically significant. Analysis will be on intention-to-treat and per-protocol subject principles. The baseline characteristics of patients in two groups will be reported. The primary outcome, relapse rate, will be compared between both groups at 12 weeks and 24 weeks after treatment using Chi-square test, and considering superiority comparison between two groups by 95% confidence interval method. The secondary outcomes will be summarized with frequency, mean, standard deviation, median, and range. At one time point, comparisons between the experiment group and the placebo group will be conducted using the t-test. In order to distinguish the treatment effect and time effect, changes from baseline in the above outcomes will be tested using repeated measure analysis of variance. The analysis of covariance or logistic regression model will be used to analyze the site effect and impact factors. The co-variables will include gender, age, concomitant drugs, disease course, BSA, and PASI at the baseline visit. The subgroup analysis will be performed based on the severity of disease and CM patterns. Adjusting for baseline covariates, for timed endpoints such as time to relapse and time to onset, we will use the Kaplan-Meier survival analysis followed by the multivariable Cox proportional hazards model for adjusting for baseline variables. To assess the impact of potential clustering for patients cared for by the same hospital, we will use generalized estimating equations (GEE) assuming an exchangeable correlation structure.

Safety will be evaluated by tabulations of adverse events and will be presented with descriptive statistics at baseline and follow-up visits for each group. The statistics will be organized by treatment phase and post-treatment phase as appropriate. Chi-square test or Fisher’s exact test will be used to compare the frequency difference of adverse events between the experimental and control group. As cases are divided into different degrees of AE, the rank-sum test will be performed for analyzing the independent ordered multiple category data between two groups.

## Discussion

Chinese herbs have been widely used in China and other Asian countries over years. Simultaneously, dozens of Chinese herbal studies *in vivo* or *vitro* were conducted to figure out the ingredients or pharmacological mechanisms. In a rice model, oral administration of *Sarcandra Glabra* extract (5-caffeoylquinic acid, 3-caffeoylquinic acid, isofraxidin and so on) could alleviate the stress-induced reduction of the number of lymphocytes, the balance of CD4 + T/CD8 + T and natural killer cell activity per spleen [19]. Niu *et al*. [20] examined that arthritic mice treated with isofraxidin (IF) had an obvious difference in serum tumor necrosis factor-α (TNF-α) compared with the lipopolysaccharides-stimulated group, IF may possess significant anti-inflammatory activities. Astilbin isolated from *Rhizoma Smilacis Glabrae*, inhibited the footpad swelling, arthritic incidence, and clinical scores without losing body weight [21]. The previous pharmacological properties of *Sarcandra Glabra* extract or *Rhizoma Smilacis Glabrae* may at least partially explain the clinical benefits for the YXBCM01 formula.

The inhibitory effect of the YXBCM01 formula has also been shown on the epithelial cell mitosis of mice [22]. Furthermore, the effect on proliferating cell nuclear antigen (PCNA) presentation and keratinocytes (KC) apoptosis was studied through different dose lavage in mice, in which high dose had a remarkable influence on PCNA inhibition and COLO-16 apoptosis [23]. Also, the YXBCM01 decoction was found to significantly inhibit the mitosis of mouse vaginal epithelium and promote the formation of granular layers in mouse tail-scale epidermis, and inhibit human keratinocyte line HaCat cells growth remarkably, when compared with the saline control group [24]. Among the five main constituents in the YXBCM01 formula, isofraxidin was found to be the active constituent for its correlation with the pathogenesis of psoriasis, with astilbin as the helper constituent due to its relationship with the transportation of drug molecules [25].

Some studies have shown that CM agents are effective and safe in the treatment of psoriasis. A randomized trial was conducted to compare CM combined with an acitretin capsule with CM alone [8]; the adverse reaction of the acitretin capsule could be alleviated by adjusting the herbs used. In another randomized control trial, 78 patients were randomly assigned to two groups: oral CM decoction (30 patients), or compound amino-polypeptide tablet (28 patients) [26]. At 4 weeks, there was an increase significantly between CM group (83.33%) and control group (64.28%) in the total response rate of PASI. Quality of life improvement in the CM group was superior to that the control. Similarly, Gao and Xu [27] demonstrated that a compound amino-polypeptide tablet combined with a CM decoction (94.29%) had an advantage over amino-polypeptide (83.33%) or CM decoction alone (62.86%) in PASI reduction. The incidence of adverse events in the CM group was less than that in amino-polypeptide and integrated group, as well as the relapse rate. However, the research quality was low and the definition of relapse was not declared. Remission seems to be long lasting and less costly compared with other treatments or biologic agents [28], but the relapse rate of CM treatment has not been proved in randomized controlled trials before.

A pilot study is necessary and important to demonstrate the feasibility of this trial, which has been performed to inform the design, from August 2012 to July 2013 in Guangzhou, China. It used the same add-on designed, double-blinded, randomized controlled trial comparing the YXBCM01 granule to placebo. The results showed the relapse rate in the YXBCM01 granule group was 28.6% (2 out of 7 patients) versus 70% (7 out of 10 patients) in the placebo group. While the result was not against our supposition of sample size calculation, we revised some details in the study design after this pilot study and it paved the way for patient recruitment and trial conducting.

As a randomized controlled trial of CM for psoriasis, it is unusual that both the CM granule and placebo groups receive topical therapy treatment. We aim to conduct this trial to demonstrate both the efficacy and safety of the YXBCM01 formula for PV patients, and consider that a conscientiously performed trial and effective outcomes will benefit PV patients. If this trial provides high-quality evidence for the efficacy and safety of the YXBCM01 formula, it will provide useful clinically information for PV, especially for reducing the relapse of disease in PV patients.

### Trial status

The pilot phase of the trial started in August 2012 in the Guangdong Provincial Hospital of Chinese Medicine, China and is still in progress.

## Abbreviations

AEs: Adverse events; BSA: Body surface area; CM: Chinese Medicine; DLQI: The Dermatology Life Quality Index; GMP: Good manufacturing practice; GPHCM: Guangdong Provincial Hospital of Chinese Medicine; IF: Isofraxidin; IWRS-CMT: The Interactive Web Response System for Chinese Medicine Trials3; KC: Keratinocytes; KUMCR: The Key Unit of Methodology in Clinical Research; PASI: The Psoriasis Area Severe Index; PASI-50: PASI score decreases more than 50% from base-line; PCNA: Proliferating cell nuclear antigen; PUVA: Psoralen combined with ultraviolet A; PV: Psoriasis vulgaris; SAS: Self-rating anxiety scale; SDS: Self-rating depression scale; SF-36: The MOS item short form health survey; TNF-α: Tumor necrosis factor-α; UVB: Ultraviolet radiation B; VAS: Visual analogue scale.

## Competing interest

The authors declare they have no competing interests.

## Authors’ contributions

ZHW and MLX: manuscript writing. MLX, DNY, YHY and CJL: data collection and critical revision. CJL and ZHW: conception and design, analysis, and final approval of the manuscript. XYL, GL and XFG: design, critical revision. AHO: data analysis, critical revision. All authors read and approved the final manuscript.

## Supplementary Material

Additional file 1 Study period and registration information. Click here for file

Additional file 2 List of approvals from 6 hospitals’ ethics committees and archival filing management required in 5 hospitals’ ethics committees. Click here for file

## References

[B1] Parisi R Symmons DP Griffiths CE Ashcroft DM Global epidemiology of psoriasis: a systematic review of incidence and prevalence J Invest Dermatol 2013 133 377 385 23014338 10.1038/jid.2012.339

[B2] Ding X Wang T Shen Y Wang X Zhou C Tian S Liu Y Peng G Zhou J Xue S Wang R Tang Y Meng X Pei G Bai Y Liu Q Zhang J Prevalence of psoriasis in China: a population-based study in six cities Eur J Dermatol 2012 22 663 667 22910173 10.1684/ejd.2012.1802

[B3] Shao C Zhang G Bao Y Jiang Z Han G Gu H National epidemiological investigation team of psoriasis of China The national psoriasis epidemiological survey in China J Dermatol Venereol 1989 1 60 71

[B4] Chen Z Zhang JZ Topical application of psoriasis vulgaris Chin J Drug Applic Monitor 2005 1 34 37

[B5] Nast A Boehncke WH Mrowietz U Ockenfels HM Philipp S Reich K Rosenbach T Sammain A Schlaeger M Sebastian M Sterry W Streit V Augustin M Erdmann R Klaus J Koza J Muller S Orzechowski HD Rosumeck S Schmid-Ott G Weberschock T Rzany B Deutsche Dermatologische Gesellschaft (DDG), Berufsverband Deutscher Dermatologen (BVDD) S3- Guidelines on the treatment of psoriasis vulgaris (English version). Update J Dtsch Dermatol Ges 2012 10 Suppl 2 S1 S95 10.1111/j.1610-0387.2012.07919.x 22386073

[B6] Puig L Bordas X Carrascosa JM Dauden E Ferrandiz C Hernanz JM Lopez-Estebaranz JL Moreno JC Sanchez-Carazo JL Vanaclocha F Vazquez-Veiga H Grupo Espanol de Psoriasis de la Academia Espanola de Dermatologia y Venereologia Consensus document on the evaluation and treatment of moderate-to-severe psoriasis. Spanish psoriasis group of the Spanish Academy of Dermatology and Venereology Actas Dermosifiliogr 2009 100 277 286 19463230

[B7] Lin YK Wong WR Chang YC Chang CJ Tsay PK Chang SC Pang JH The efficacy and safety of topically applied indigo naturalis ointment in patients with plaque-type psoriasis Dermatology 2007 214 155 161 17341866 10.1159/000098576

[B8] Zhang LX Bai YP Song PH You LP Yang DQ Effect of Chinese herbal medicine combined with acitretin capsule in treating psoriasis of blood-heat syndrome type Chin J Integr Med 2009 15 141 144 19407953 10.1007/s11655-009-0145-5

[B9] Zhou N Bai YP Man XH Zhang YB Kong YH Ju H Chang M Effect of new Pulian Ointment in treating psoriasis of blood-heat syndrome: A randomized controlled trial Chin J Integr Med 2009 15 409 414 20082244 10.1007/s11655-009-0409-0

[B10] Zhang JW Wang JZ Zhang H Pang CK Liu YX The evidence-based evaluation of the randomized controlled trials of Chinese herbal treatment of the psoriasis vulgaris China J Leprosy Skin Dis 2003 19 370 372

[B11] Deng S May BH Zhang AL Lu C Xue CC Topical herbal formulae in the management of psoriasis: systematic review with meta-analysis of clinical studies and investigation of the pharmacological actions of the main herbs Phytother Res 2014 28 480 497 23817996 10.1002/ptr.5028

[B12] Deng S May BH Zhang AL Lu C Xue CC Topical herbal medicine combined with pharmacotherapy for psoriasis: a systematic review and meta-analysis Arch Dermatol Res 2013 305 179 189 23354931 10.1007/s00403-013-1316-y

[B13] Yan YH Lu CJ Optimized Yinxieling for treatment of psoriasis vulgaris: an exploratory clinical trial Zhong Yao Xin Yao Yu Lin Chuang Yao Li 2011 22 691 931

[B14] Menter A Gottlieb A Feldman SR Van Voorhees AS Leonardi CL Gordon KB Lebwohl M Koo JY Elmets CA Korman NJ Beutner KR Bhusha R Guidelines of care for the management of psoriasis and psoriatic arthritis: Section 1. Overview of psoriasis and guidelines of care for the treatment of psoriasis with biologics J Am Acad Dermatol 2008 58 826 850 18423260 10.1016/j.jaad.2008.02.039

[B15] Psoriasis Study Group, skin venereal diseases branch of Chinese Medical Association Guideline for the treatment of psoriasis (2008) Chin J Dermatol 2009 3 213 214

[B16] Gordon KB Feldman SR Koo JY Menter A Rolstad T Krueger G Definitions of measures of effect duration for psoriasis treatments Arch Dermatol 2005 141 82 84 15655149 10.1001/archderm.141.1.82

[B17] Carey W Glazer S Gottlieb AB Lebwohl M Leonardi C Menter A Papp K Rundle AC Toth D Relapse, rebound, and psoriasis adverse events: an advisory group report J Am Acad Dermatol 2006 54 S171 S181 16488339 10.1016/j.jaad.2005.10.029

[B18] White S Vender R Thaci D Haverkamp C Naeyaert JM Foster R Martinez Escribano JA Cambazard F Bibby A Use of calcipotriene cream (Dovonex cream) following acute treatment of psoriasis vulgaris with the calcipotriene/betamethasone dipropionate two-compound product (Taclonex): a randomized, parallel-group clinical trial Am J Clin Dermatol 2006 7 177 184 16734505 10.2165/00128071-200607030-00004

[B19] He RR Yao XS Li HY Dai Y Duan YH Li YF Kurihara H The anti-stress effects of Sarcandra glabra extract on restraint-evoked immunocompromise Biol Pharm Bull 2009 32 247 252 19182384 10.1248/bpb.32.247

[B20] Niu X Xing W Li W Fan T Hu H Li Y Isofraxidin exhibited anti-inflammatory effects in vivo and inhibited TNF-alpha production in LPS-induced mouse peritoneal macrophages in vitro via the MAPK pathway Int Immunopharmacol 2012 14 164 171 22800929 10.1016/j.intimp.2012.06.022

[B21] Cai Y Chen T Xu Q Astilbin suppresses collagen-induced arthritis via the dysfunction of lymphocytes Inflamm Res 2003 52 334 340 14504671 10.1007/s00011-003-1179-3

[B22] Lu CJ Liu FL The effect of Yinxieling tablet on epithelial cell mitosis Zhong Guo Lin Chuang Yi Xue 2007 19 25 26

[B23] Lu CJ Wu XX Liu FL Effect of PCNA presentation and KC apoptosis of Yinxieling Zhong Yao Xin Yao Yu Lin Chuang Yao Li 2006 17 329 331

[B24] Han L Yu JH Lou BQ Zhao RZ Lu CJ BIBM committee: IEEE Computer Society Effects of Yinxieling-Optimization-Decoction in treating psoriasis in mouse psoriasis models Proceedings of the 2011 IEEE International Conference on Bioinformatics and Biomedicine Workshops: 12-15 November 2011; Atlanta 2011 Washington DC, USA 807 810

[B25] Zhu W He SM Yuan XH Lu CJ Computerized Systematic Pharmacological Research of Yinxieling Formula Zhong Yao Xin Yao Yu Lin Chuang Yao Li 2011 22 379 382

[B26] Li FL Li B Xu R Song X Yu Y Xu ZC Qinzhu Liangxue Decoction in treatment of blood-heat type psoriasis vulgaris: a randomized controlled trial Zhong Xi Yi Jie He Xue Bao 2008 6 586 590 18559235 10.3736/jcim20080608

[B27] Gao BA Xu X Assessment on effect of treatment of chronic plaque type psoriasis by combination therapy of composite shendi decoction and diyin tablet Zhongguo Zhong Xi Yi Jie He Za Zhi 2001 21 15 18 12577369

[B28] Wang G Liu Y Traditional Chinese medicine is effective and safe in the treatment of psoriasis Int J Dermatol 2004 43 552 15230903 10.1111/j.1365-4632.2004.02181.x

