# Erratum to: 'Chinese medicine combined with calcipotriol betamethasone and calcipotriol ointment for Psoriasis vulgaris (CMCBCOP): study protocol for a randomized controlled trial'

**DOI:** 10.1186/s13063-016-1500-4

**Published:** 2016-07-26

**Authors:** Ze-Huai Wen, Mei-Ling Xuan, Yu-Hong Yan, Xiao-Yan Li, Dan-Ni Yao, Geng Li, Xin-Feng Guo, Ai-Hua Ou, Chuan-Jian Lu

**Affiliations:** 1 Key Unit of Methodology in Clinical Research, Guangdong Provincial Hospital of Chinese Medicine, 111 Dade Road, Guangzhou, 510120 China; 2 National Centre for Design Measurement and Evaluation in Clinical Research, Guangzhou University of Chinese Medicine, 12 Jichang Road, Guangzhou, 510405 China; 3 Department of Dermatology, Guangdong Provincial Hospital of Chinese Medicine, 111 Dade Road, Guangzhou, 510120 China

## Erratum

Unfortunately, the original version of this article [1] contained an error. The superiority margin difference should be 5 % instead of 10 %. We performed a superiority test of two independent proportions with superiority margin difference of 5 % by PASS 11.0 software again, and the results were the same as mentioned in the paper before. The sentence in the "Sample size calculation" part (Page 5) is corrected as follows:

According to the supposition and as calculated by PASS 11.0 software (NCSS, LLC, Kaysville, Utah, USA), sample size of 239 in each group can achieve 90 % power and to rule out a two-sided type I error of 5 % to detect a superiority margin difference of 5 % in this two-arm trial with equal allocation in each group.
