# Supplementary material for: Chinese medicine combined with calcipotriol betamethasone and calcipotriol ointment for Psoriasis vulgaris (CMCBCOP): study protocol for a randomized controlled trial
Source: Trials. 2014 Jul 22;15:294. doi: 10.1186/1745-6215-15-294 (PMC4223761; doi:10.1186/1745-6215-15-294)
Supplement: Additional file 1 — Study period and registration information. [file 1745-6215-15-294-S1.doc]

|  | Study period | | | | | | | | | | | | |
| --- | --- | --- | --- | --- | --- | --- | --- | --- | --- | --- | --- | --- | --- |
|  | enrolment | allocation | Post-allocation | | | | | | | Close-out | Follow-up | | |
| TIMEPOINT | -2w | 0 | 1w | 2w | 3w | 4w | 6w | 8w | 10w | 12w | 16w | 20w | 24w |
| ENROLMENT: |  |  |  |  |  |  |  |  |  |  |  |  |  |
| Eligibility screen | X |  |  |  |  |  |  |  |  |  |  |  |  |
| Run-in period | X |  |  |  |  |  |  |  |  |  |  |  |  |
| Informed consent | X |  |  |  |  |  |  |  |  |  |  |  |  |
| characteristic | X |  |  |  |  |  |  |  |  |  |  |  |  |
| Medical history | X |  |  |  |  |  |  |  |  |  |  |  |  |
| Lab tests |  | X |  |  |  | X |  |  |  | X |  |  |  |
| Biological specimens |  | X |  |  |  | X |  |  |  | X |  |  |  |
| Allocation |  | X |  |  |  |  |  |  |  |  |  |  |  |
| INTERVENTIONS: |  |  |  |  |  |  |  |  |  |  |  |  |  |
| *YXBCM01 granule*plus topical treatment |  |  |  |  |  |  |  |  |  |  |  |  |  |
| Placebo plus topical treatment |  |  |  |  |  |  |  |  |  |  |  |  |  |
| ASSESSMENT: |  |  |  |  |  |  |  |  |  |  |  |  |  |
| CM syndrome |  | X |  |  |  | X |  |  |  | X |  |  |  |
| PASI | X | X | X | X | X | X | X | X | X | X | X | X | X |
| SAS |  | X |  |  |  |  |  |  |  |  |  |  |  |
| SDS |  | X |  |  |  |  |  |  |  |  |  |  |  |
| VAS |  | X | X | X | X | X | X | X | X | X | X | X | X |
| DLQI |  | X |  |  |  | X |  | X |  | X |  |  | X |
| SF-36 |  | X |  |  |  | X |  | X |  | X |  |  | X |
| Adverse event |  |  | X | X | X | X | X | X | X | X | X | X | X |
| Combination |  | X | X | X | X | X | X | X | X | X | X | X | X |

Additional file 1

Registration data

| **Data category** | **Information** |
| --- | --- |
| Primary registry and trial identifying number | ChiCTR-TRC-13003233 |
| Date of registration in primary registry | 26 May, 2013 |
| Secondary identifying numbers | U1111-1151-1332 |
| Source(s) of monetary or material support | The Ministry of Science and Technology of the People's Republic of China |
| Primary sponsor(s) | Guangdong Provincial Hospital of Chinese Medicine |
| Contact for public queries | Chuanjian Lu, MD, luchuanjian888@vip.sina.com |
| Contact for scientific queries | Chuanjian Lu, MD, Guangdong Provincial Hospital of Chinese Medicine, Guangdong, China |
| Public title | Clinical study according to pathogenesis treatment for psoriasis vulgaris |
| Scientific title | Chinese Medicine Combined with Calcipotriol Betamethasone and Calcipotriol Ointment for Psoriasis Vulgaris: A Double-blind Randomised Controlled Trial |
| Countries of recruitment | China |
| Health condition(s) or problem(s) studied | Chinese medicine, *YXBCM01 granule*, psoriasis vulgaris |
| Intervention (s) | Active comparator: *YXBCM01 granule*(11g per day, 12 weeks) plus Calcipotriol Betamethasone and Calcipotriol Ointment |
|  | Placebo comparator: placebo granules (matching granules containing no active ingredients) plus Calcipotriol Betamethasone and Calcipotriol Ointment |
| Key inclusion and exclusion criteria | Ages eligible for study: ≥ 18 years; Sexes eligible for study: both; Accepts healthy volunteers: no |
|  | Inclusion criteria: adult patient (≥ 18 years), stationary stage, PASI> 10 or BSA > 10, meanwhile PASI<30 and BSA<30, inform consent. |
|  | Exclusion criteria: Guttate psoriasis, inverse psoriasis or exclusively involves the face; pregnant, lactating, or which one plan to become pregnant in a year; SAS > 50 or SDS > 53, or with other psychiatric disorders; history of cardiovascular, respiratory, digestive, urinary, and hematologic disease, which can’t controlled through common treatment, either with cancer, infection, electrolyte imbalance, acid-base disturbance and calcium metabolic disorder; allergic to any medicine or ingredients used in this study; participating other clinical trials or participated within 1 month; topical treatments (i.e. corticosteroids, Retinoic acid) within 2 weeks; systemic therapy or phototherapy (UVB and PUVA) within 4 weeks; biological therapy within 12 weeks; acute progression of psoriasis, and erythroderma tendency; systemic treatment prescribed by doctors. |
| Study type | Interventional |
|  | Allocation: randomized; Intervention model: parallel assignment; Masking: double blind |
|  | Primary purpose: therapy |
|  | Method of sequence generation and allocation concealment: |
| Date of first enrollment | Oct 2013 |
| Target sample size | 600 |
| Recruitment status | Recruiting |
| Primary outcome(s) | Relapse rate, Psoriasis Area and Severity Index score |
| Key secondary outcomes | Relapse time interval, onset time, rebound rate, cumulative consumption of medicine, Visual Analogue Scale, the Body Surface Area, the Dermatology Quality Life Index, the MOS item short form health survey |
