# Supplementary material for: Chinese medicine combined with calcipotriol betamethasone and calcipotriol ointment for Psoriasis vulgaris (CMCBCOP): study protocol for a randomized controlled trial
Source: Trials. 2014 Jul 22;15:294. doi: 10.1186/1745-6215-15-294 (PMC4223761; doi:10.1186/1745-6215-15-294)
Supplement: Additional file 2 — List of approvals from 6 hospitals’ ethics committees and archival filing management required in 5 hospitals’ ethics committees. [file 1745-6215-15-294-S2.doc]

Additional file 2

List of approvals from 6 hospitals’ ethics committees and archival filing management required in 5 hospitals’ ethics committees

1. Institutional Ethics Committee of Guangdong Provincial Hospital of Traditional Chinese Medicine (approval reference: B2012-53-01, and accelerated review approval reference B2012-53-03 for version 002/201302)

2. Ethics Committee of China-Japan Friendship Hospital (approval reference: 2013-17 )

3. Ethics Committee of Longhua Hospital, Shanghai University of TCM (approval reference: 2013LCSY024 )

4. Ethics Committee of Xinjiang Uygur Autonomous Region Hospital of Traditional Chinese Medical (approval reference: 2013XE016 )

1. Ethics Committee of the First Affiliated Hospital of Guangzhou University of Chinese Medicine (approval reference: [2013]020)
2. Sichuan Regional Ethics Review Committee on Traditional Chinese Medicine (approval reference: 2013KL-024)

Approval document for Affiliated Hospital of Chengdu University of Chinese Medicine

1. Ethics Committee of Guang’anmen Hospital, China Academy of Chinese Medical Sciences

Required archival filing management

1. Ethics Committee of Beijing Hospital of Traditional Chinese Medicine

Required archival filing management

1. Ethics Committee of Heilongjiang Academy of Traditional Chinese Medicine

Required archival filing management

1. Ethics Committee of Wuhan Integrated Traditional Chinese Medicine & Western Medicine Hospital

Required archival filing management

1. Ethics Committee of the Third Hospital of Hangzhou

Required archival filing management
